# CT-based prediction of *EGFR* mutation subtypes and *TP53* co-mutation status in lung adenocarcinoma

**DOI:** 10.3389/fmolb.2026.1898841

**Published:** 2026-10-21

**Authors:** Haixian Liu, Zhi Li, Yingqi Zhang, Guohua Yu, Yaqi Miao, Xiaotong Teng, Yueli Xu, Wenyuan Xu, Chengpeng Gao, Fan Bai, Lun Li

**Affiliations:** 1 Respiratory and Critical Care Medicine Center, Weifang People’s Hospital, Weifang, China; 2 National Clinical Research Center for Medical Auxiliary Technology(Laboratory Medicine), Department of Laboratory Medicine, The First Hospital of China Medical University, Shenyang, China; 3 Research Unit of Medical Laboratory, Chinese Academy of Medical Sciences, Shenyang, China; 4 School of Mechanical and Automation, Weifang University, Weifang, China; 5 Shandong Key Laboratory of Intelligent Manufacturing, Weifang, China; 6 Department of Pathology, Yantai Yuhuangding Hospital, Yantai, China; 7 Department of Pulmonary and Critical Care Medicine, Affiliated Hospital of Shandong Second Medical University, Weifang, China; 8 Department of Pulmonary and Critical Care Medicine, Shandong Provincial Chest Hospital, Jinan, China; 9 Department of Pulmonary and Critical Care Medicine, The Second Affiliated Hospital of Shandong First Medical University, Tai’an, China; 10 School of Equipment Engineering, Shenyang Ligong University, Shenyang, China

**Keywords:** chest CT, deep learning, *EGFR* mutation subtype, lung adenocarcinoma, *TP53* co-mutation

## Abstract

**Background:**

Epidermal growth factor receptor (*EGFR*) mutation status is a key determinant of targeted treatment in lung adenocarcinoma, whereas co-occurring *TP53* alterations may further affect therapeutic response and prognosis. This study aimed to develop a noninvasive CT-based approach for predicting *EGFR* mutation subtypes and *TP53* co-mutation status in lung adenocarcinoma, thereby providing imaging support for pretreatment risk stratification and clinical decision-making.

**Methods:**

Patients with pathologically confirmed lung adenocarcinoma who underwent pretreatment chest CT and molecular testing were retrospectively enrolled from a single-center cohort. A total of 1,633 patients were included and categorized into five molecular groups: *EGFR* wild type, *EGFR* 19del, *EGFR* L858R, *EGFR* 19del with *TP53* co-mutation, and *EGFR* L858 R with *TP53* co-mutation. Based on chest CT images, key slice extraction, lesion ROI generation, automatic segmentation, and image standardization were performed. Semantic CT features, radiomics features, and deep CT representations were then derived. Under a unified data partitioning and standardized evaluation framework, four models were constructed and compared for the five-class classification task: a random forest model based on semantic CT features, a support vector machine model based on radiomics features, a Qwen-based semantic classification model, and a MedicalDINOv3-based deep learning model. To eliminate confounding bias induced by multimodal feature fusion, two ablation experiments were supplemented to split the model input features, and two MedicalDINOv3 models were built with inputs restricted to semantic features only and CT images only, respectively. Model performance was assessed using accuracy, macro-averaged area under the receiver operating characteristic curve (AUC), and macro-F1 score. External validation was further conducted using public and multicenter datasets.

**Results:**

Among the 1,633 patients in the internal cohort, 731 were *EGFR* wild type, 295 had *EGFR* 19del, 399 had *EGFR* L858R, 118 had *EGFR* 19del with *TP53* co-mutation, and 90 had *EGFR* L858 R with *TP53* co-mutation. To quantify the independent predictive value of different input modalities, ablation experiments on three variants of MedicalDINOv3 were performed. Ablation results revealed distinct performance among three input strategies. The MedicalDINOv3 with only semantic input obtained a test accuracy of 31.55% and an AUC of 0.6305 with limited predictive ability. The branch solely fed with CT images reached an accuracy of 55.61% and a macro-average AUC of 0.6347. The complete MedicalDINOv3 integrating CT deep features, clinical and semantic features performed best, with a test accuracy of 0.6905 and a macro-average AUC of 0.8788. This comparison proved that CT deep representations outperform traditional handcrafted imaging and semantic features, and multi-modal fusion further improves subtype stratification performance. The support vector machine model achieved an accuracy of 0.527, a macro-AUC of 0.792 and a macro-F1 score of 0.524; the random forest model achieved an accuracy of 0.455, a macro-AUC of 0.819 and a macro-F1 score of 0.412; and the Qwen-based model achieved an accuracy of 0.339, a macro-AUC of 0.700 and a macro-F1 score of 0.316. Receiver operating characteristic analyses showed that the deep learning model provided relatively stable discrimination for *EGFR* wild type and *TP53* co-mutated categories, whereas overlap remained between the two major *EGFR*-sensitive subtypes, *EGFR* 19del and *EGFR* L858 R. In external validation, the public dataset used for *EGFR* binary classification yielded an overall accuracy of 67.0% and an AUC of 0.6666. The raw overall accuracy of the cohort was 75.5%, and raw overall accuracy is prone to overestimation when the cohort has severe class imbalance dominated by *EGFR* wild-type samples, which can only serve as an auxiliary reference. Multicenter five-class external validation yielded a balanced accuracy of 58.2% and a macro F1 of 0.574. The subtype-specific AUCs for *EGFR* wild type, *EGFR* 19del, *EGFR* L858R, *EGFR* 19del with *TP53* co-mutation, and *EGFR* L858 R with *TP53* co-mutation were 0.8342, 0.6504, 0.7221, 0.6586, and 0.7563, respectively.

**Conclusion:**

A CT-based deep learning model can provide noninvasive stratification of EGFR mutation subtypes and TP53 co-mutation status in lung adenocarcinoma. Compared with semantic CT features and radiomics features, deep CT representations showed superior overall discriminative performance. This approach may serve as an adjunctive tool to molecular testing for pretreatment assessment in lung adenocarcinoma. However, the model showed inconsistent generalization performance across different molecular subtypes, with notably limited predictive efficacy for EGFR 19del and EGFR 19del+TP53 subtypes. Therefore, predictions for these categories should be interpreted with caution, and the model requires further validation in larger, multicenter, and standardized datasets before clinical application.

## 1 Background

Lung adenocarcinoma is one of the most common histological subtypes of non-small cell lung cancer. Epidermal growth factor receptor (*EGFR*) mutations, particularly exon 19 deletions and exon 21 L858 R substitutions, are among the most important actionable molecular alterations in lung adenocarcinoma and serve as key determinants for tyrosine kinase inhibitor treatment (Haixian et al., 2025; Li Y et al., 2025). With the widespread use of *EGFR* tyrosine kinase inhibitors, *EGFR* mutation status has become a central biomarker for precision stratification in lung adenocarcinoma. However, the presence of an *EGFR* mutation does not necessarily imply uniform clinical benefit. Differences in biological behavior and treatment response have been observed across *EGFR* mutation subtypes, and some patients with sensitizing mutations still experience primary resistance or early disease progression. In recent years, *TP53* co-mutation has received increasing attention (Miyawaki et al., 2025). Previous studies have suggested that, compared with patients harboring *EGFR* mutations alone, those with concurrent *TP53* alterations tend to have a higher risk of progression and poorer clinical outcomes. Therefore, further identification of *TP53* co-mutation status on the basis of *EGFR* mutation may allow more refined risk stratification and provide additional support for individualized treatment planning (Li S et al., 2025).

At present, tissue biopsy and molecular testing remain the standard approaches for determining *EGFR* and *TP53* status. However, these methods still face several practical limitations in routine clinical settings. First, tissue sampling is invasive, and adequate specimens may be difficult to obtain in some patients because of lesion location, clinical condition, or sample constraints. Second, a single biopsy may not fully reflect intratumoral spatial heterogeneity, and the results may therefore be influenced by sampling bias. In addition, molecular testing is associated with turnaround time, cost, and limited feasibility for repeated dynamic assessment. In this context, the development of a noninvasive, repeatable, and clinically accessible auxiliary approach is of clear practical value (Unger and Kather, 2024; Boehm et al., 2022). Chest computed tomography is one of the most commonly used imaging modalities for lung cancer screening, diagnosis, and staging, and is routinely available in clinical practice. It can simultaneously capture multiple levels of imaging information, including tumor morphology, attenuation, lesion margins, and surrounding pulmonary changes, thereby providing an important basis for noninvasive prediction of tumor molecular phenotypes (Wang S et al., 2022).

In recent years, CT-based radiomics and deep learning approaches have shown promise for predicting *EGFR* mutation status in lung adenocarcinoma (Kather et al., 2020; Zhao et al., 2025). However, most previous studies have mainly focused on the binary classification of *EGFR*-mutant versus *EGFR*-wild-type tumors (Huang et al., 2025; Xu J et al., 2025; Xu S et al., 2025). Reports on the discrimination of major sensitizing *EGFR* subtypes and the further identification of *TP53* co-mutation remain limited, which restricts their utility for more refined clinical stratification (Deng et al., 2025). At the same time, many studies have primarily emphasized the tumor itself, while underusing potentially informative imaging cues derived from the lesion boundary, adjacent lung parenchyma, and the surrounding pulmonary environment (Bai et al., 2026). Accordingly, there remains a need for CT-based models that are better aligned with clinically relevant molecular stratification (Wu et al., 2023; Zeng et al., 2023; Wang X et al., 2022; Moon et al., 2023).

Based on these considerations, the present study used pretreatment chest CT as the core imaging source and constructed a prediction framework for five molecular categories: *EGFR* wild type, *EGFR* 19del, *EGFR* L858R, *EGFR* 19del with *TP53* co-mutation, and *EGFR* L858 R with *TP53* co-mutation. Unlike previous studies that mainly addressed binary *EGFR* classification, this study further compared the performance of semantic CT features, radiomics features, and deep CT representations in a five-class classification task, and incorporated external validation to assess model stability and generalizability. Through this work, we aimed to evaluate the potential value of routine chest CT for molecular stratification in lung adenocarcinoma and to explore its feasibility as an adjunctive tool for pretreatment risk assessment and supplementary molecular evaluation.

## 2 Methods

### 2.1 Study population

This was a single-center retrospective study. We retrospectively collected data from patients with lung cancer who underwent *EGFR*-related molecular testing at Weifang People’s Hospital between January 2018 and December 2025. After case verification, a traceable base cohort of 7,229 patients was established, with a relatively balanced distribution of *EGFR*-mutant and *EGFR*-wild-type cases and a subset of patients harboring concomitant genomic alterations.

The inclusion criteria were as follows: (1) pathologically confirmed lung adenocarcinoma; (2) availability of complete pretreatment chest CT images; and (3) definite molecular pathology results that allowed determination of *EGFR* and *TP53* status. The exclusion criteria were as follows: (1) receipt of antitumor treatment before imaging and molecular testing; (2) an interval of more than 1 month between CT examination and pathological or molecular testing; (3) missing key clinical or imaging data; (4) severe pleural effusion affecting lesion assessment; and (5) multiple primary lung cancers with inconsistent *EGFR* status across lesions.

The patient selection workflow is shown in Figure 1. A total of 13,206 testing records were initially identified. After data verification, 7,229 traceable cases were retained. Patients who did not meet the pathological and prespecified study criteria were then excluded step by step, resulting in a final cohort of 1,633 patients with lung adenocarcinoma for analysis. Standardized imaging, molecular, and clinical data from these patients were used for subsequent model development and validation.

**FIGURE 1:**
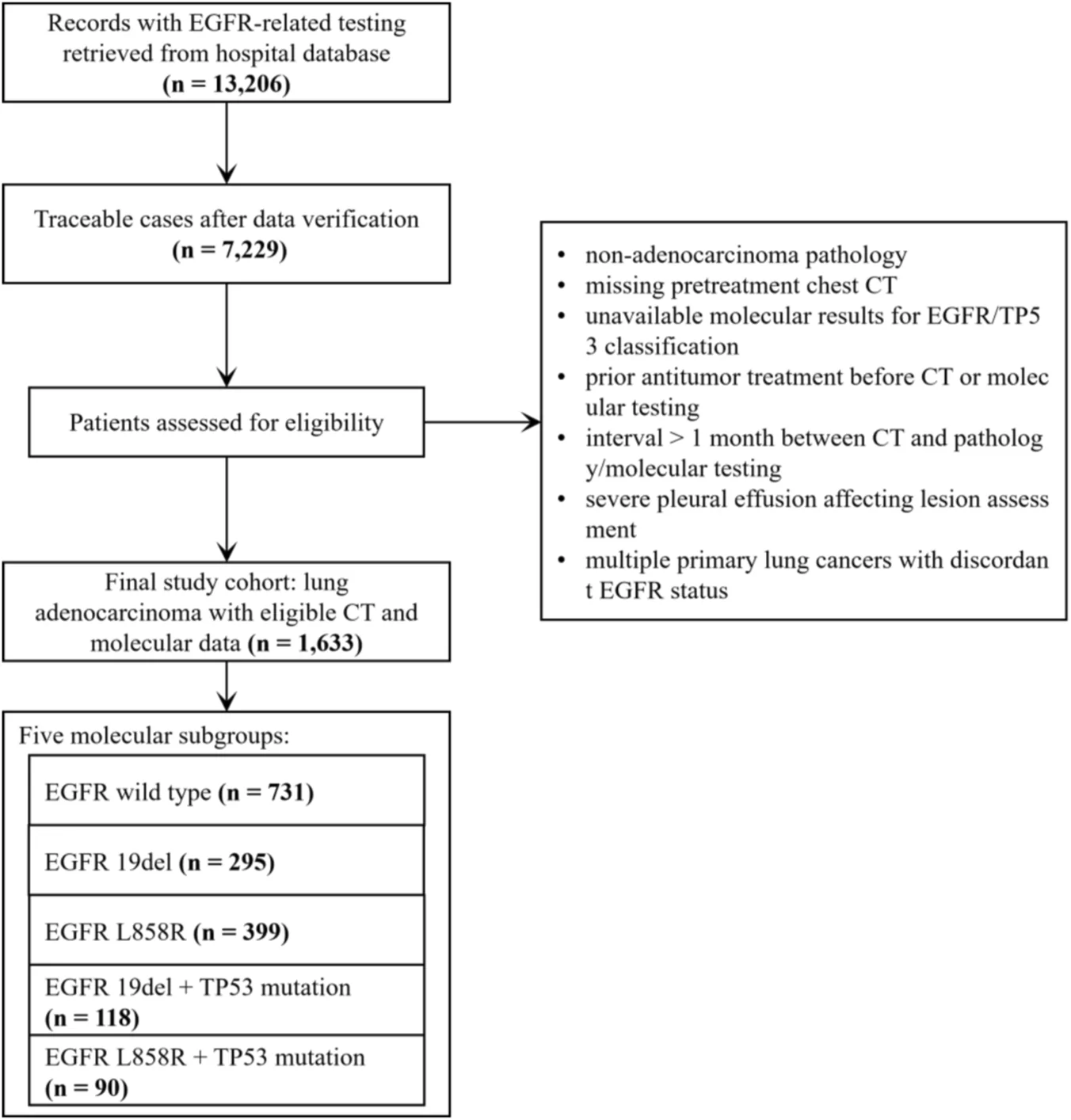
Patient selection workflow for the study cohort. A total of 13,206 testing records were initially retrieved. After data verification and sequential application of the eligibility criteria, 1,633 patients with lung adenocarcinoma were finally included in the analysis and categorized into five molecular subgroups.

Molecular status was determined according to the reports issued by the Molecular Pathology Center of Weifang People’s Hospital. Testing methods included both single-gene *EGFR* assays and multigene panel testing. Formalin-fixed paraffin-embedded tissue specimens were used in most cases, while cell blocks prepared from cytological samples were used when tissue samples were insufficient or clinically required. All molecular tests were performed as part of routine clinical care and were reviewed according to laboratory quality-control procedures before inclusion in the analysis.

According to *EGFR* and *TP53* mutation status, the final study cohort was categorized into five molecular subgroups: *EGFR* wild type, *EGFR* 19del, *EGFR* L858R, *EGFR* 19del with *TP53* mutation, and *EGFR* L858 R with *TP53* mutation. Because the primary aim of this study was to evaluate stratification across *EGFR* sensitizing subtypes and *TP53* co-mutation, *TP53* status was not further subdivided in the *EGFR*-wild-type group. This asymmetric grouping incorporates patients carrying both wild-type and mutant *TP53* into the *EGFR* wild-type group, raising intra-group imaging phenotypic heterogeneity and partially blurring the classification boundaries between wild-type cases and each mutant subtype. When interpreting model performance, the discriminative power for the wild-type class represents the capacity to separate the entire *EGFR* wild-type population from all *EGFR* mutant subgroups, instead of precise identification of the pure population with both wild-type *EGFR* and wild-type *TP53*. The numbers of patients in the five subgroups were 731, 295, 399, 118, and 90, respectively.

To systematically evaluate the generalization robustness of this multi-modal stratification prediction model across diverse populations and medical centers, two mutually independent external validation cohorts were constructed in this study to test model performance. The first cohort was a public multi-omics integrated dataset combining cases from The Cancer Imaging Archive (TCIA), The Cancer Genome Atlas (TCGA), and the Clinical Proteomic Tumor Analysis Consortium (CPTAC) databases (Clark et al., 2013; Heath et al., 2021). This dataset predominantly consists of European and American patient samples, and only provides binary labeling of *EGFR* wild-type/mutant status without complete molecular annotations for *EGFR* 19del, L858 R subtypes and *TP53* co-mutations. Thus, it was only applicable to external validation of binary *EGFR* classification.

The public access links for the databases are listed as follows: TCIA (https://wiki.cancerimagingarchive.net), TCGA (https://www.cancer.gov/ccg/research/genome-sequencing/tcga), and CPTAC (https://pdc.cancer.gov/pdc/browse).

The second cohort was a self-established retrospective multicenter external cohort from Shandong Province, which consecutively enrolled lung adenocarcinoma patients from five Grade A tertiary hospitals: the Affiliated Hospital of Shandong Second Medical University, Yantai Yuhuangding Hospital, Shandong Public Health Medical Center, Tai’an No.1 People’s Hospital, and Shanxi Provincial Hospital of Traditional Chinese Medicine. All enrolled patients were matched with complete pretreatment chest CT images, whole-slide digital H&E pathology sections, standardized clinical baseline data, and five-category molecular reports of *EGFR/TP53* detected via NGS or ARMS-PCR, enabling external validation and analysis for the full five molecular subtypes (Figure 2).

**FIGURE 2:**
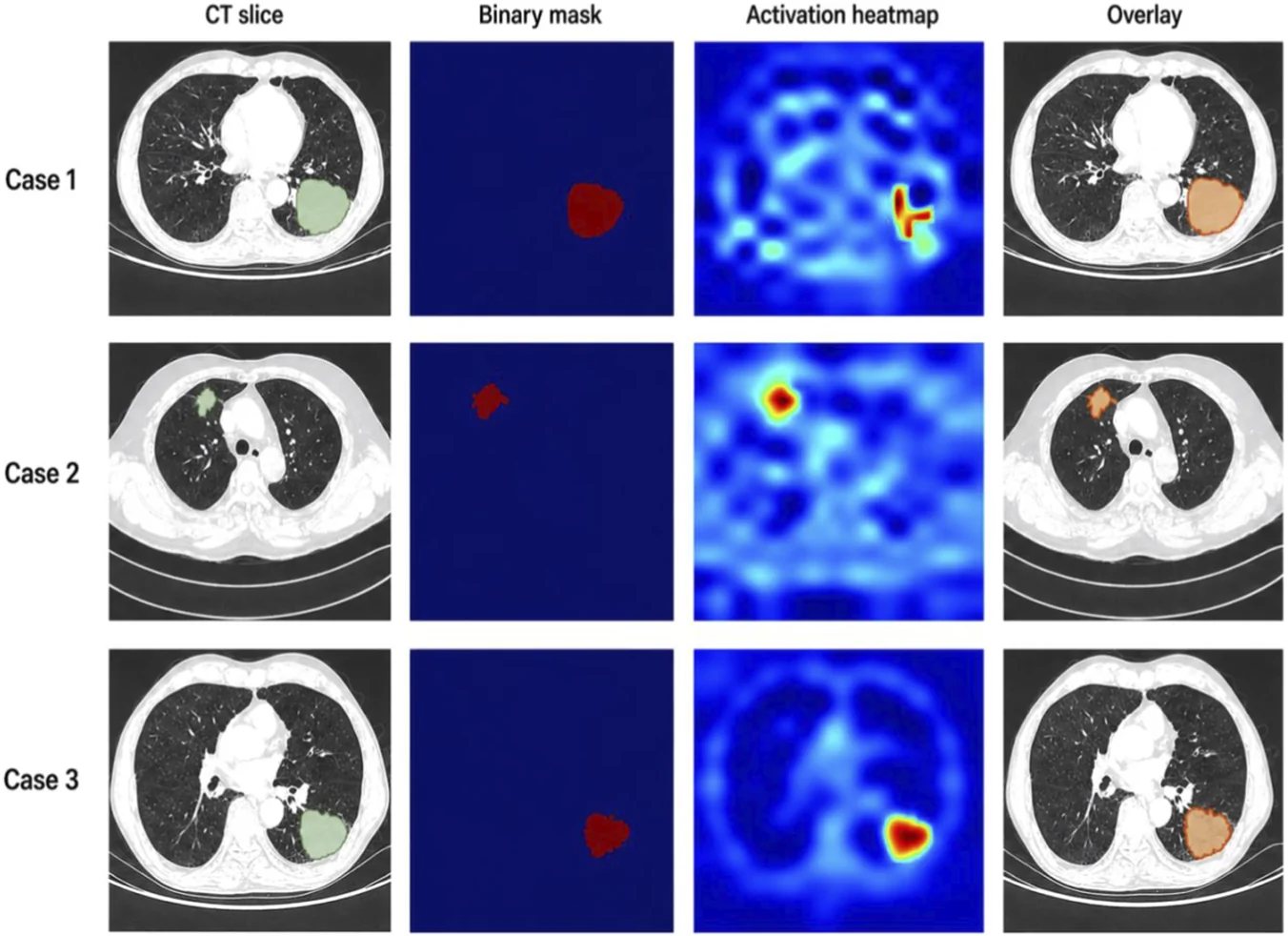
Representative lesion-level visualization of CT model activation: Three representative cases are shown. For each case, the columns display the original CT slice, binary lesion mask, activation heatmap and lesion overlay. Warmer colors in the activation heatmap indicate regions with higher model response. The high-response regions were spatially aligned with the segmented tumor regions across lesions with different locations and sizes, supporting the lesion-centered nature of the learned CT representations.

### 2.2 CT acquisition and baseline CT selection

Chest CT images used in this study were retrieved from the Picture Archiving and Communication System of Weifang People’s Hospital. All lung CT examinations were performed using Siemens CT scanners, with scan coverage extending from the lung apex to the lung base. The main acquisition parameters included a tube voltage of 120 kV, tube current of 200–500 mA, rotation time of 0.4–0.7 s, a reconstruction matrix of 512 × 512, and reconstruction kernels of B31f or B30f. Imaging data were extracted on a case-by-case basis from the hospital system using IQQA-Chest software and exported in DICOM format. All images were de-identified before further analysis.

To maximize temporal consistency between imaging features and molecular results, prespecified rules were applied for baseline CT selection. Baseline CT was defined as the chest CT examination used for clinical assessment before treatment initiation or tissue sampling, and scans obtained within 7 days before planned treatment were preferentially selected. If both non-contrast and contrast-enhanced CT scans were available within the same time window, the contrast-enhanced examination was preferentially used. For patients without CT available at the predefined time point, a chest CT acquired after diagnosis but before treatment could be used as a substitute, provided that it covered the entire thorax and included adequate thin-section series. Examinations that did not meet the study consistency or image quality criteria were excluded from subsequent analysis.

### 2.3 CT semantic feature assessment

CT examinations used for semantic feature assessment were the baseline chest CT scans obtained closest to the time of pathological sampling and molecular testing within the same pretreatment clinical stage. Imaging review was independently performed by three thoracic radiologists using the PACS workstation, with 5, 8, and 13 years of experience, respectively. To minimize subjective bias, the readers were blinded to the molecular classification of all patients, and the case order was randomized before statistical analysis. In cases of disagreement, a consensus interpretation was reached through discussion. To assess the inter-rater consistency of semantic feature interpretation, repeated evaluations were performed on 60 randomly selected cases. High inter-observer agreement was achieved (ICC >0.90), verifying the stability of semantic feature extraction.

Semantic CT features were recorded according to a prespecified assessment form and mainly included lesion location, primary morphologic appearance, margin characteristics, shape, enhancement pattern, and common associated imaging signs. Specifically, lesion location was recorded by pulmonary lobe or hilar/central region. Primary morphologic appearance was categorized as nodule, mass, or consolidation. Margins were classified as well-defined or ill-defined/blurred, and shape was recorded as regular/round or irregular. Enhancement pattern was classified as homogeneous enhancement or heterogeneous enhancement/necrosis. Additional semantic features included ground-glass opacity components, air bronchogram, bubble-like lucency or cavitation, lobulation, spiculation, pleural retraction, vessel convergence, vascular involvement, and pleural effusion.

The definitions of these semantic features followed routine thoracic radiologic descriptors. Ground-glass opacity was defined as a ground-glass attenuation component within or around the lesion. Air bronchogram was defined as the presence of air-filled bronchi within the lesion. Bubble-like lucency or cavitation referred to gas-containing lucent areas within the lesion. Lobulation, spiculation, pleural retraction, vessel convergence, and vascular involvement were assessed according to their typical imaging appearances. Pleural effusion was recorded as present or absent. All semantic features were organized in a structured format for subsequent statistical analysis and model development.

### 2.4 Lesion segmentation and radiomics feature extraction

After baseline CT selection and semantic feature assessment, lesion regions were further segmented in a standardized manner, and radiomics features were subsequently extracted from the segmentation results. The overall workflow included lesion ROI generation, automatic segmentation, image intensity standardization, segmentation quality control, and radiomics feature extraction. The main steps of this procedure are illustrated in Figures 3, 4.

**FIGURE 3:**
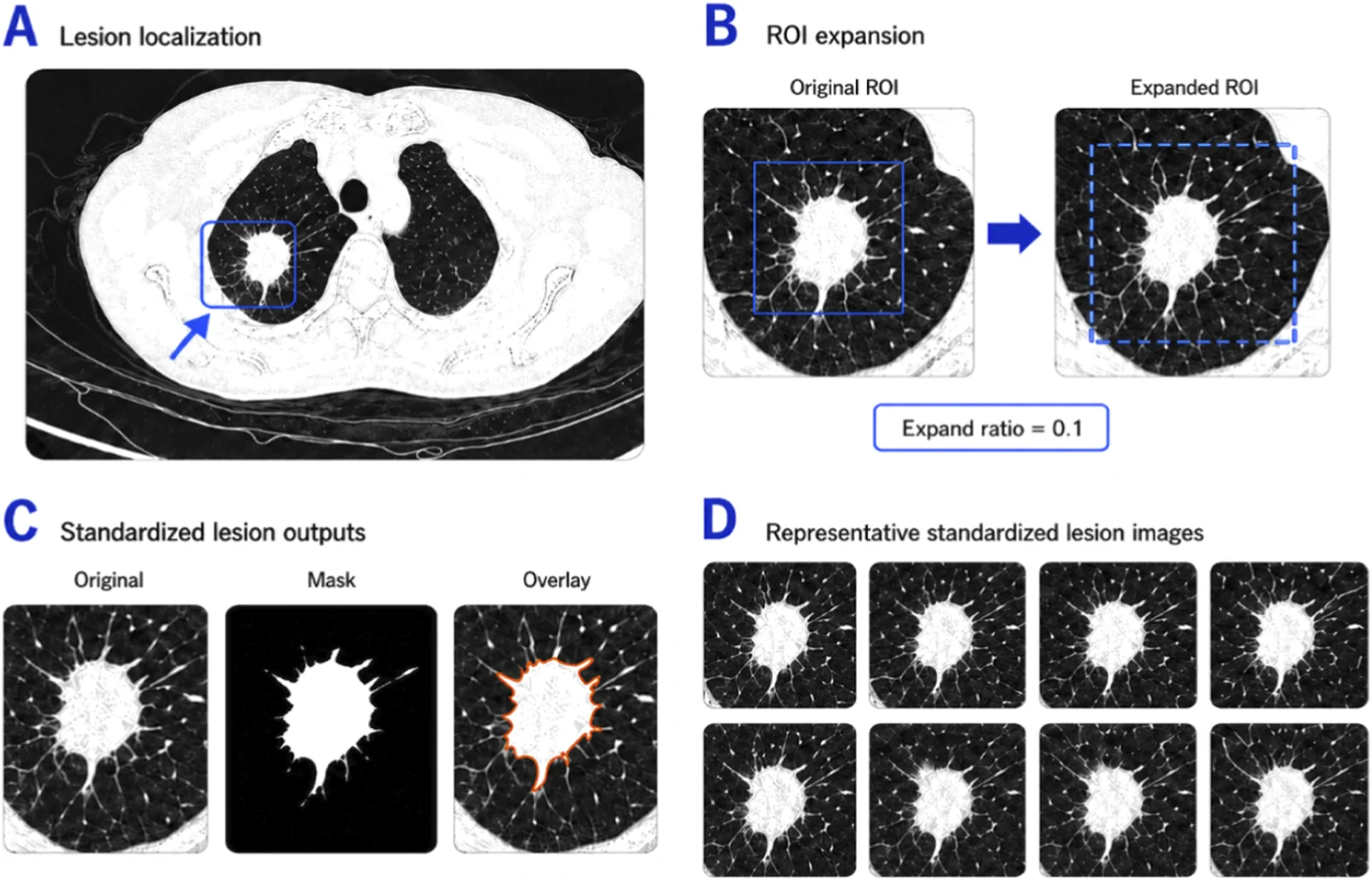
CT lesion localization and standardized image preparation: **(A)** Representative lesion localization on pretreatment chest CT; **(B)** Lesion-centred ROI expansion; **(C)** Standardized outputs including the original ROI, binary mask and overlay image; **(D)** Representative standardized lesion images used for downstream analysis.

**FIGURE 4:**
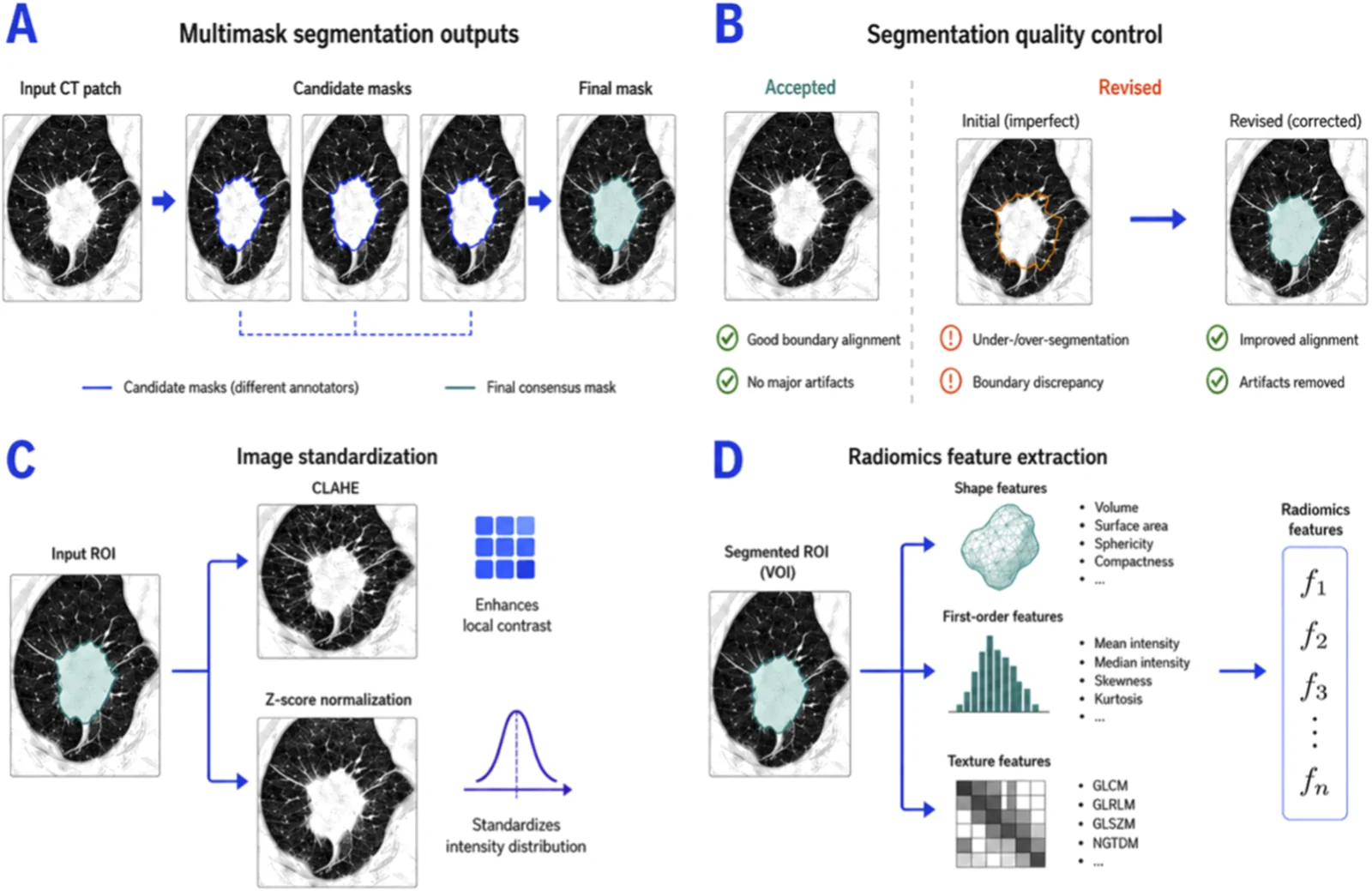
Segmentation quality control and radiomics feature extraction: **(A)** Multimask segmentation outputs for a representative CT patch; **(B)** Segmentation quality control of accepted and revised masks; **(C)** Image standardization using CLAHE and Z-score normalization; **(D)** Radiomics feature extraction from the segmented ROI/VOI.

Specifically, lesion ROIs were generated on the basis of lesion bounding boxes exported from the workstation. To preserve relevant information from the tumor margin and adjacent lung parenchyma, the initial lesion region was moderately expanded before segmentation. Balancing margin preservation against background noise control, the ROI expansion ratio was set to 0.1. The lesion localization and ROI generation process is shown in Figures 3A,B.

Following ROI construction, lesion segmentation was independently revised and validated by two radiologists with over 5 years of thoracic imaging diagnostic experience based on automated segmentation outputs. Initial segmentation masks were automatically generated via Segment Anything Model 2 (SAM2). Featuring robust zero-shot generalization and precise boundary perception, SAM2 effectively captures grayscale density differences between lesions and adjacent lung parenchyma on CT images to achieve efficient preliminary automatic delineation of lesion contours (Ravi et al., 2024). To address cases with indistinct tumor margins or lesion adhesion to adjacent vessels or pleura, segmentation inference was conducted in multimask mode, which generated multiple candidate masks together with confidence scores. The highest-scoring candidate was selected as the primary segmentation result, while all candidate masks were retained for manual inspection and failure analysis. All mask revisions and quality control were independently performed by the two aforementioned radiologists, with standardized thoracic imaging anatomy guidelines as the uniform criterion. Cases with evident boundary deviations were manually revised or excluded. Quality control focused on completeness of lesion coverage and inadvertent inclusion of non-tumor structures such as vessels or pleura. The entire segmentation annotation and revision process was fully blinded to patients' *EGFR* and *TP53* molecular mutation status. All lesion segmentations were uniformly completed prior to dataset splitting, and no label information was involved in the manual quality control of the test set, which avoided evaluation bias introduced by awareness of group labels and ensured the reliability of performance results on the independent test set. Discrepancies between the two radiologists were resolved through joint discussion to reach a final consensus. Representative multimask segmentation outputs and quality-control examples are shown in Figures 4A,B.

To support subsequent model training, error tracing, and interpretability analysis, three standardized image outputs were saved for each lesion: the original ROI image (Original), the binary lesion mask (Mask), and the boundary overlay image (Overlay). Representative examples of these outputs are shown in Figure 3C. Segmentation-related metadata and quality-control results were written back to a structured index file to preserve lesion paths, quality-control status, and derived morphologic information.

Because CT intensity distributions may vary across acquisition conditions, contrast-limited adaptive histogram equalization (CLAHE) and Z-score intensity normalization were applied during ROI preprocessing. CLAHE was used to enhance local texture details in low-contrast lesions, whereas Z-score normalization was used to reduce intensity drift across scanners and acquisition settings. Representative standardization examples are shown in Figure 4C, (Wa et al., 2026). According to the predefined pipeline, the CLAHE clip limit was set to 2.0.

After lesion segmentation and image standardization, radiomics features were extracted from the segmented lesion ROI/volume of interest for subsequent model development. The extracted radiomics features mainly included shape features, first-order statistical features, and texture features. To ensure comparability across patients, these features were standardized before model training.

To evaluate the consistency of segmentation results, repeated annotation analysis was conducted on 50 randomly selected samples. The Dice similarity coefficient (DSC) and intraclass correlation coefficient (ICC) were calculated after the two radiologists independently completed lesion segmentation. The results showed favorable segmentation consistency, with a DSC of 0.92 and all ICC values greater than 0.90, indicating that the segmentation pipeline has high stability and reproducibility.

### 2.5 Deep CT representation learning

In addition to radiomics features, a deep learning-based CT representation pathway was established. Key lesion slices were used as the basic input units, and slice-level deep features were extracted and further aggregated into patient-level representations for downstream classification. Key lesion slices were manually selected by radiologists. The selection procedure was fully blinded to *EGFR* and *TP53* mutation status and completed prior to dataset splitting.

Each key slice was organized as a seven-channel input, including the original CT grayscale image, lesion-only image, perilesional background image, binary segmentation mask, and a three-channel overlay image (Figure 5). This input format was designed to preserve information from the tumor itself, lesion margins, and adjacent lung parenchyma within a single representation.

**FIGURE 5:**
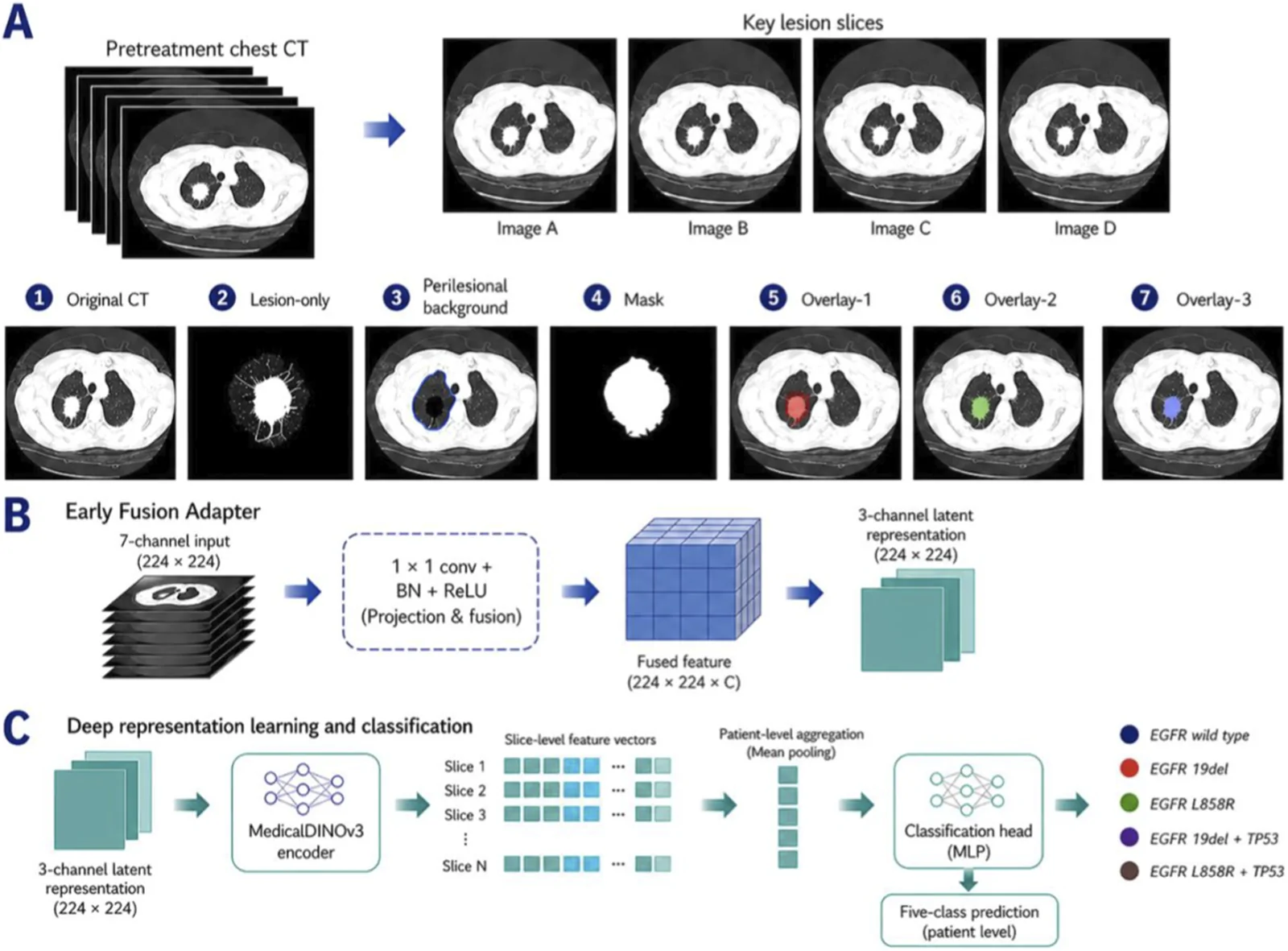
Seven-channel CT representation learning framework: **(A)** Construction of the seven-channel CT input from key lesion slices; **(B)** Projection of the seven-channel input into a three-channel latent representation through the Early Fusion Adapter; **(C)** MedicalDINOv3-based deep feature extraction, patient-level aggregation and five-class molecular subtype prediction.

Because pretrained visual models are typically designed for three-channel input, an Early Fusion Adapter was introduced to project the seven-channel input into a three-channel latent space. All input images were resized to 224 × 224, and intensity normalization was applied before network inference.

MedicalDINOv3 was then used as the core imaging encoder for deep CT representation learning. This encoder is a domain-adapted variant built upon a general pretrained visual model. Its backbone adopts the publicly available pretrained DINO v3 (ViT-L/14), with a self-developed multi-channel projection adapter and a medical feature adaptation module incorporated. The initial weights of the backbone are derived from large-scale self-supervised pretraining on natural images. A gradual unfreezing strategy is adopted during training: most parameters of the backbone remain frozen, while the last 0–6 Transformer layers can be unfrozen as required, and parameters of all newly added adaptation modules are fully updated throughout training. For patients with multiple key slices, slice-level features were further aggregated at the patient level and used for subsequent classification modeling and performance evaluation.

### 2.6 Lesion-level visualization analysis

To qualitatively assess whether the deep CT model focused on anatomically relevant lesion regions, lesion-level activation maps were generated for representative cases from the test set. For each selected case, the original CT slice, binary lesion mask, activation heatmap and lesion overlay were displayed in parallel. The activation maps were resized to the corresponding CT patch size and normalized to the range of 0–1 for visualization. A blue-to-red colour scale was used, with warmer colours indicating higher model response. Binary lesion masks and overlay images were displayed alongside the activation maps to assess the spatial consistency between model response and segmented tumor regions. This visualization analysis was used only as a qualitative interpretability aid and was not included as an independent quantitative endpoint.

### 2.7 Model development and evaluation

Given the marked imbalance in sample distribution across subtypes in the five-class classification task of this study—particularly the low prevalence of the *TP53* co-mutation subtype—tailored class imbalance mitigation strategies were implemented during the training phase to prevent model bias toward majority classes.

For the deep learning model MedicalDINOv3, inverse-frequency class weighting derived from training set class frequencies was applied, combined with Focal Loss (γ = 1.0) to enhance the model’s capacity to learn from hard-to-classify samples. For conventional machine learning models, including random forest, support vector machine, and LASSO-related baselines, random oversampling via RandomOverSampler was performed exclusively on the training set to alleviate class imbalance, while the validation and test sets retained their original sample distributions. Neither SMOTE nor generative data augmentation approaches were adopted in this study to avoid potential distribution shift.

The primary task was a five-class classification, with output categories including *EGFR* wild type, *EGFR* 19del, *EGFR* L858R, *EGFR* 19del with *TP53* mutation, and *EGFR* L858 R with *TP53* mutation. All 1,633 patients were split before model development into training, validation, and test sets at a fixed ratio of 7:2:1, corresponding to 1,143, 327 and 163 patients, respectively. The training set was used for model fitting and feature processing, the validation set was used for model selection, hyperparameter tuning and threshold setting, and the test set was reserved exclusively for final performance reporting to minimize information leakage and ensure a fair comparison across models.

Under the same data split and evaluation framework, six comparative models were constructed. A random forest model was trained using structured semantic CT features to evaluate the discriminative value of clinically interpretable imaging phenotypes. A support vector machine model was trained using radiomics features extracted from segmented lesions to assess the predictive value of quantitative texture and morphologic information. The Qwen-based semantic classification model served as a non-visual language baseline. It took only clinical information and structured semantic CT description texts as input, mapped them into a unified semantic space, and performed five-class classification by calculating similarity to predefined class description prototypes, to evaluate the discriminative capacity of language/semantic information alone for this task (Yang et al., 2025; Gao et al., 2025; Xiao et al., 2025; Dobreva et al., 2025; Wu et al., 2025). Two feature-split ablation control experiments were additionally designed based on the MedicalDINOv3 network. The first was a semantic-feature-only input ablation branch, which aimed to ascertain the upper limit of classification performance achievable by pure semantic signs under the identical deep learning architecture. The second was a pure CT deep representation ablation branch, in which clinical data and structured semantic features were completely excluded, to independently quantify the inherent discriminative efficacy of CT deep representations and address the limitation that performance contributions cannot be traced in multi-feature fusion scenarios. A MedicalDINOv3-based fusion model was constructed by concatenating patient-level deep CT representations with selected clinical and semantic CT features, followed by a multilayer perceptron classifier for five-class prediction.

Feature preprocessing, standardization, and necessary feature selection were performed within the training set only. For radiomics features, dimensionality reduction and feature selection were restricted to the training data. For the deep learning model, the validation set was used to monitor training and determine early stopping. All models generated five-class predictions and corresponding scores in the test set for subsequent performance comparison.

Model performance was evaluated in terms of both discrimination and classification. Discrimination was assessed using one-vs-rest area under the receiver operating characteristic curve for each class, together with macro-averaged AUC. Classification performance was mainly evaluated using accuracy, precision, recall, and macro-F1 score, with class-specific metrics reported when needed. All model training, prediction, and visualization were performed in Python.

### 2.8 External validation cohorts

External validation was performed using two independent datasets. The public dataset included 66 patients with available CT images and binary *EGFR* mutation labels, including 19 *EGFR*-mutant and 47 *EGFR*-wild-type cases (Clark et al., 2013; Heath et al., 2021). Because detailed *EGFR* subtype and *TP53* co-mutation labels were not consistently available, this dataset was used only for binary *EGFR* classification. The multicenter validation cohort included 116 patients with complete five-class molecular labels, including 71 *EGFR* wild type, 18 *EGFR* 19del, 15 *EGFR* L858R, 7 *EGFR* 19del with *TP53* co-mutation, and 5 *EGFR* L858 R with *TP53* co-mutation. External CT images were processed using the same lesion localization, ROI generation, segmentation, image standardization and model inference pipeline as the internal cohort. Model parameters were fixed during external validation, and no additional training or fine-tuning was performed on the external datasets.

### 2.9 Statistical analysis

Baseline clinical variables and CT semantic features were summarized and compared across the five molecular subgroups. Continuous variables with an approximately normal distribution were expressed as mean ± standard deviation and compared using one-way analysis of variance. Non-normally distributed continuous variables were expressed as median with interquartile range and compared using the Kruskal–Wallis test. Categorical variables were presented as counts and percentages and compared using the Pearson χ^2^ test; Fisher’s exact test was used when expected cell counts were small. All statistical tests were two-sided, and P < 0.05 was considered statistically significant. Baseline statistical analyses were performed using SPSS version 22.0.

Model performance was evaluated on the independent test set, and the same evaluation framework was applied to the external validation cohorts. For the five-class classification task, discrimination was assessed using a one-vs-rest strategy to calculate the area under the receiver operating characteristic curve for each class, together with macro-averaged AUC. Classification performance was mainly evaluated using accuracy, precision, recall, and macro-F1 score. Class-specific metrics were reported when needed to reflect performance balance across molecular subtypes. Model results were summarized using confusion matrices, ROC curves, and external validation results.

All preprocessing, feature selection, hyperparameter tuning, and threshold setting related to model development were restricted to the training and validation sets, whereas the test set was reserved exclusively for final performance reporting to minimize information leakage. Model training, prediction, and visualization were performed in Python.

## 3 Results

### 3.1 Patient characteristics

A total of 1,633 patients with lung adenocarcinoma were included in the study. The cohort was predominantly middle-aged and older, with a mean age of 63.75 ± 10.24 years (range, 14–92 years), and showed a nearly balanced sex distribution, with 801 men and 832 women. Never-smokers accounted for the largest proportion of patients (72.4%). Advanced-stage disease was more common, with stage IV accounting for 58.7% of the cohort. Regarding specimen source, percutaneous lung biopsy was the most common type (54.1%), followed by surgical specimens and effusion cytology. The main baseline characteristics are summarized in Table 1.

**TABLE 1:** Baseline clinical characteristics stratified by molecular subtype.

Characteristic	Overall (n = 1,633)	Wild type (n = 731)	19del (n = 295)	L858 R (n = 399)	19del+*TP53* (n = 118)	L858 R+*TP53* (n = 90)	P value
Age, years	63.75 ± 10.24	63.63 ± 10.97	62.77 ± 9.40	64.79 ± 9.79	61.16 ± 9.39	66.68 ± 8.62	<0.001
Sex							<0.001
Male	801 (49.1%)	456 (62.4%)	123 (41.7%)	133 (33.3%)	52 (44.1%)	37 (41.1%)	
Female	832 (50.9%)	275 (37.6%)	172 (58.3%)	266 (66.7%)	66 (55.9%)	53 (58.9%)	
Smoking status							<0.001
Never smoker	1,183 (72.4%)	472 (64.6%)	225 (76.3%)	323 (81.0%)	91 (77.1%)	72 (80.0%)	
Former smoker	355 (21.7%)	213 (29.1%)	54 (18.3%)	55 (13.8%)	18 (15.3%)	15 (16.7%)	
Current smoker	95 (5.8%)	46 (6.3%)	16 (5.4%)	21 (5.3%)	9 (7.6%)	3 (3.3%)	
Clinical stage							<0.001
I–III	674 (41.3%)	394 (53.9%)	95 (32.2%)	130 (32.6%)	25 (21.2%)	30 (33.3%)	
IV	959 (58.7%)	337 (46.1%)	200 (67.8%)	269 (67.4%)	93 (78.8%)	60 (66.7%)	

Data are presented as mean ± standard deviation or n (%), unless otherwise indicated. The complete stratified baseline table is provided in Supplementary Table S1.

According to the prespecified molecular classification, 731 patients were categorized as *EGFR* wild type, 295 as *EGFR* 19del, 399 as *EGFR* L858R, 118 as *EGFR* 19del with *TP53* mutation, and 90 as *EGFR* L858 R with *TP53* mutation. Overall, *EGFR*-related molecular subtypes accounted for 55.2% of the study cohort, and 23.1% of patients with *EGFR* mutations harbored concurrent *TP53* alterations. Among imaging-related clinical manifestations, lymph node metastasis was the most common finding, followed by bone metastasis, intrapulmonary metastasis, and malignant pleural effusion. The full stratified clinical statistics are provided in Supplementary Table S1.

### 3.2 Comparison of model performance in the five-class classification task

Under the same data split and evaluation framework, four models, including MedicalDINOv3, support vector machine, random forest, and Qwen, were compared in the five-class classification task for *EGFR/TP53* stratification in lung adenocarcinoma. To further trace the source of model performance gains and eliminate confounding interference from multi-feature fusion, single-feature modality ablation validations were performed in parallel to investigate the independent classification efficacy of pure semantic features and pure CT deep representations, respectively. Overall, the MedicalDINOv3 model achieved the best performance, with a test-set accuracy of 0.701, a macro-averaged AUC of 0.879, and macro-precision, macro-recall, and macro-F1 values of 0.693, 0.691, and 0.690, respectively (Table 2). Notably, macro-averaged AUC is calculated as the equal-weight average of per-class AUC values. It can therefore partially mitigate the impact of class distribution imbalance on overall evaluation; however, disparities in sample proportions across categories may still cause fluctuations in the local performance assessment of different subtypes.

**TABLE 2:** Overall performance of different models in the five-class classification task.

Model	Accuracy	Macro-AUC	Macro-precision	Macro-recall	Macro-F1
MedicalDINOv3 fusion model	0.701	0.879	0.693	0.691	0.690
MedicalDINOv3-CT only	0.556	0.635	0.399	0.393	0.387
MedicalDINOv3-clinical text only	0.315	0.630	0.286	0.281	0.262
SVM	0.527	0.792	0.529	0.527	0.524
Random forest	0.455	0.819	0.658	0.455	0.412
Qwen	0.339	0.700	0.402	0.339	0.316

Data are reported for the independent test set. Macro-AUC, macro-precision, macro-recall, and macro-F1, were calculated across the five molecular subtypes.

In comparison, the support vector machine model achieved an accuracy of 0.527, a macro-AUC of 0.792, and a macro-F1 score of 0.524. The random forest model achieved an accuracy of 0.455, a macro-AUC of 0.819, and a macro-F1 score of 0.412, whereas the Qwen-based model achieved an accuracy of 0.339, a macro-AUC of 0.700, and a macro-F1 score of 0.316 (Table 2). The Qwen-based semantic classification model served as a language baseline relying exclusively on structured semantic information. This result indicates that text semantic information alone cannot fully capture the spatial and textural features embedded in CT images. Although the random forest model retained a certain degree of discriminative ability in terms of macro-AUC, its lower accuracy and macro-F1 score suggested limited stability for practical five-class classification. Focusing on the single-modality ablation results of MedicalDINOv3, the model fed with only structured semantic features showed severely impaired classification performance, with an accuracy of merely 31.55% and a macro-AUC as low as 0.6305. Its performance was lower than that of the baseline models in several evaluation metrics, suggesting that pure semantic features alone have limited discriminative capacity for molecular subtyping. In contrast, the single-branch model that extracted deep representations exclusively from CT images, without integration of clinical or semantic information, achieved an accuracy of 55.61% and a macro-AUC of 0.6347. The CT-only branch performed better than the semantic-feature-only branch and the Qwen-based semantic baseline, suggesting that pixel-level CT information provided additional discriminative value beyond structured semantic descriptions. However, it did not consistently outperform the radiomics-based SVM model across all metrics, as its macro-AUC and macro-F1 were lower despite a slightly higher accuracy.

Taken together, the MedicalDINOv3 model based on deep CT representations showed the highest numerical performance among the evaluated approaches in both overall discrimination and classification performance, and was therefore selected as the primary candidate model for subsequent class-specific analysis and external validation.

### 3.3 Class-specific discrimination across the five molecular subtypes

To further evaluate class-specific discrimination, receiver operating characteristic curves were generated using a one-vs-rest strategy for *EGFR* wild type, *EGFR* 19del, *EGFR* L858R, *EGFR* 19del + *TP53*, and *EGFR* L858 R + *TP53* (Figure 6), and the corresponding AUC values were calculated (Supplementary Table S2). Overall, the ROC curves of MedicalDINOv3 were consistently closer to the upper-left corner across the five molecular subtypes, indicating better and more balanced discrimination across classes. In comparison, the support vector machine and random forest models retained a certain degree of class-specific discrimination in some subtypes but showed greater variability, whereas the Qwen-based model showed overall weaker performance. Ablation analysis revealed that the semantic-only branch achieved consistently inferior performance across molecular subtypes. Specifically, the *EGFR* L858 R + *TP53* subtype yielded an AUC of only 0.4904, falling below the performance of conventional machine learning baselines, including random forest and SVM, suggesting that semantic descriptors alone lack sufficient discriminative power for identifying *TP53* co-mutational patterns. In contrast, the CT image-only branch demonstrated strong predictive capability for the *EGFR* wild-type subtype, achieving an AUC of 0.8821. The *EGFR* L858 R single-mutation subtype also exhibited reliable discrimination (AUC = 0.8069) and outperformed the *EGFR* 19del subtype (AUC = 0.6759). Nevertheless, both *TP53* co-mutated subtypes showed limited performance in the image-only setting (AUC <0.41), markedly inferior to that of the full multimodal fusion model. These results highlight that accurate discrimination of *TP53* co-mutated subtypes requires complementary clinical and semantic information beyond CT imaging features alone.

**FIGURE 6:**
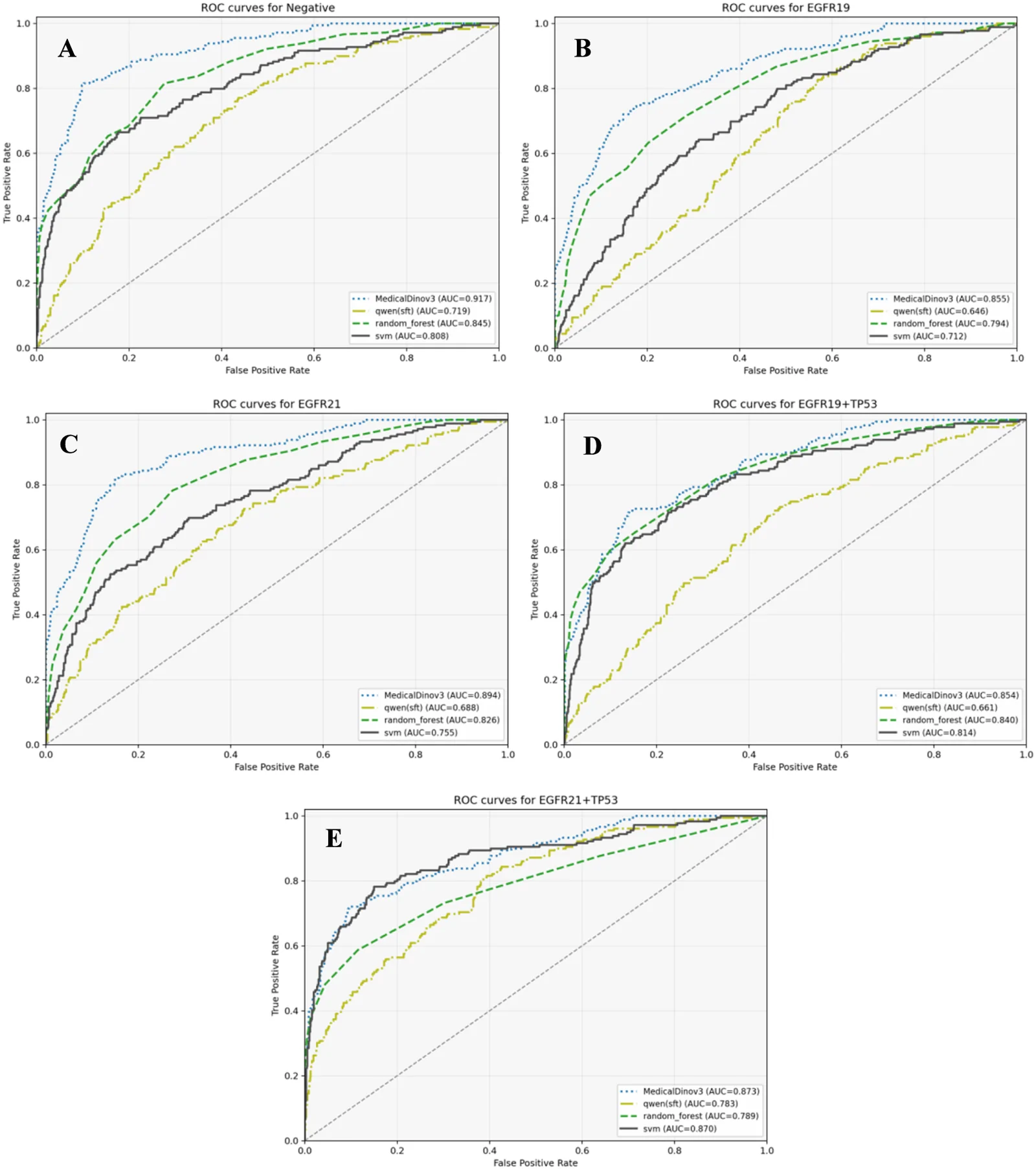
Receiver operating characteristic curves of the four models for the five molecular subtypes. **(A)** *EGFR* wild type; **(B)** *EGFR* 19del; **(C)** *EGFR* L858R; **(D)** *EGFR* 19del + *TP53*; **(E)** *EGFR* L858 R + *TP53*.

Among the five subtypes, discrimination was most stable for the *EGFR*-wild-type group, for which MedicalDINOv3 achieved an AUC of 0.917, compared with 0.845 for random forest, 0.808 for support vector machine, and 0.719 for Qwen. For the two major sensitizing *EGFR* subtypes, the AUCs of MedicalDINOv3 were 0.855 for *EGFR* 19del and 0.894 for *EGFR* L858R, both higher than those of the other models, although some overlap between these two classes remained. For the *TP53* co-mutated categories, MedicalDINOv3 also maintained favorable discrimination, with AUCs of 0.854 for *EGFR* 19del + *TP53* and 0.873 for *EGFR* L858 R + *TP53*. Notably, the support vector machine model achieved AUCs of 0.814 and 0.870 in these two co-mutated categories, respectively, suggesting some value in identifying *TP53*-related subgroups; however, its overall stability and class balance remained inferior to those of MedicalDINOv3.

MedicalDINOv3 showed favorable discrimination across *EGFR* wild type, *EGFR* single-mutation, and *TP53* co-mutated categories, with relatively limited fluctuation across classes. These findings were consistent with the overall performance comparison and further supported the advantage of deep CT representations for five-class *EGFR/TP53* stratification in lung adenocarcinoma. More detailed class-wise classification metrics are provided in the supplementary materials.

### 3.4 Qualitative visualization of lesion-level model activation

Representative visualization maps were generated to assess whether the deep CT model captured lesion-relevant information. In three representative cases, the activation heatmaps showed high-response regions that were spatially aligned with the segmented tumor masks and overlay regions on the corresponding CT slices (Figure 2). This localization pattern was observed in lesions with different sizes and anatomical locations, suggesting that the model primarily relied on tumor-centered and peritumoral imaging cues rather than unrelated background signals. These qualitative findings support the interpretability of the seven-channel CT representation and provide visual evidence for lesion-level feature learning.

### 3.5 External validation performance

To evaluate model generalizability, external validation was performed using two independent datasets, including a public dataset for binary *EGFR* classification and a multicenter cohort for five-class classification (Figure 7). The public dataset included 66 patients with available CT images and binary *EGFR* mutation labels, including 19 *EGFR*-mutant and 47 *EGFR*-wild-type cases. Because detailed *EGFR* subtype and *TP53* co-mutation labels were not consistently available, this dataset was used only for binary *EGFR* classification. The multicenter validation cohort included 116 patients with complete five-class molecular labels, including 71 *EGFR* wild-type cases, 18 *EGFR* 19del cases, 15 *EGFR* L858 R cases, 7 *EGFR* 19del with *TP53* co-mutation cases, and 5 *EGFR* L858 R with *TP53* co-mutation cases.

**FIGURE 7:**
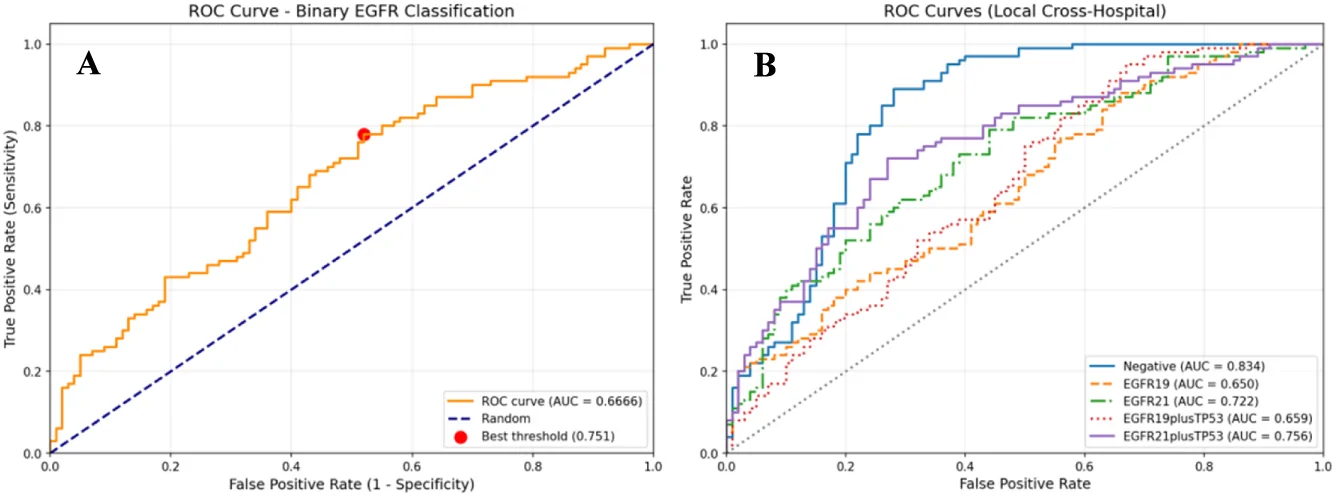
External validation performance of the CT-based model: **(A)** Receiver operating characteristic curve for binary *EGFR* classification in the public dataset. **(B)** Receiver operating characteristic curves for five-class classification in the multicenter cohort.

For binary *EGFR* classification in the TCIA-TCGA-CPTAC combined public dataset, the model achieved an overall accuracy of 67.0% and an AUC of 0.6666 (Table 3; Figure 7A). The ROC curve remained above the diagonal reference line, indicating that the model retained a certain degree of discrimination across externally sourced data. However, its performance was lower than that observed in the internal test set, suggesting reduced generalizability under different data distributions and imaging conditions.

**TABLE 3:** External validation performance of the CT-based model.

Validation cohort	Classification task	Metric	Value
Public dataset (N = 66)	*EGFR* binary classification	Accuracy	0.670
		AUC	0.6666
Multicenter cohort (N = 116)	Five-class classification	Accuracy	0.755
		Balanced accuracy	0.582
		Macro-F1	0.574
		Macro-AUC	0.7243
		AUC (*EGFR* wild type)	0.8342
		AUC (*EGFR* 19del)	0.6504
		AUC (*EGFR* L858 R)	0.7221
		AUC (*EGFR* 19del+*TP53*)	0.6586
		AUC (*EGFR* L858 R+*TP53*)	0.7563

External validation was performed using a public dataset for binary *EGFR*, classification and a multicenter cohort for five-class classification. For the multicenter cohort, AUC, values were calculated using a one-vs-rest strategy.

For external five-class validation in the multicenter cohort, the model achieved an overall accuracy of 75.5%. The balanced accuracy was 58.2%, and the macro-averaged F1-score was 0.574. Although the raw overall accuracy was 75.5%, this metric should be interpreted with caution because it was substantially inflated by class imbalance, with the *EGFR* wild-type subgroup accounting for 61% of the cohort, and therefore may not accurately reflect the model’s true stratification performance or allow fair cross-model comparison. Using a one-vs-rest strategy, the AUCs were 0.8342 for *EGFR* wild type, 0.6504 for *EGFR* 19del, 0.7221 for *EGFR* L858R, 0.6586 for *EGFR* 19del + *TP53*, and 0.7563 for *EGFR* L858 R + *TP53* (Table 3; Figure 7B). Based on the ROC curves, discrimination was most stable for the *EGFR*-wild-type subgroup, whose curve was closest to the upper-left corner. Favorable discrimination was also observed for the *EGFR* L858 R + *TP53* subtype. In contrast, the curves for *EGFR* 19del and *EGFR* 19del + *TP53* were closer to the diagonal line, indicating relatively limited discrimination for these categories in the external cohort. These results suggest that insufficient generalizability in discriminating *EGFR* 19del and *EGFR* 19del + *TP53* co-mutated subtypes remains a major bottleneck of the current model.

These external validation results suggest that the model retained a certain degree of stratification capability across multicenter and externally sourced data, particularly for identifying *EGFR*-wild-type tumors and some high-risk co-mutated subgroups. Nevertheless, the model showed a marked decline in discriminative performance for *EGFR* 19del and *EGFR* 19del + *TP53* subtypes in external datasets, indicating insufficient generalizability for these clinically relevant molecular categories. Given the current performance limitations, the model may not yet be suitable for independent clinical decision-making. However, generalizability remained limited across different *EGFR* sensitizing subtypes, especially *EGFR* 19del-related categories, indicating that further optimization with larger, multicenter, and more standardized datasets is still needed (Figure 7).

## 4 Discussion

To address the clinical need for precise stratification of *EGFR* mutation subtypes and concurrent *TP53* mutations in lung adenocarcinoma, this study developed a five-class prediction model based on pretreatment chest CT. Under a unified evaluation framework, the MedicalDINOv3 model, driven by deep CT representations, achieved the best overall performance among the evaluated models. Its accuracy, macro-average AUC, and macro-average F1 score were numerically higher than those of support vector machine (SVM), random forest (RF), and Qwen models. To more precisely trace the core contributors to model performance improvement, we performed unimodal ablation experiments by decomposing the input feature dimensions and independently assessing the discriminative value of each feature modality for subtype classification. The results demonstrated that the MedicalDINOv3 model based solely on semantic features showed markedly degraded classification performance, rendering it inadequate for precise five-class stratification. By contrast, a model constructed using CT deep features alone, with both clinical and semantic information removed, was still able to maintain stable subtype prediction performance. This finding indicates that for complex molecular subtyping tasks, deep representation learning directly derived from CT images can extract more robust discriminative information than traditional semantic features or handcrafted radiomics (Lin et al., 2025; Lu et al., 2025).

This performance advantage is largely attributable to the multi-dimensional CT input strategy employed in our study. Diverging from conventional approaches that rely solely on the tumor entity or a single region of interest (ROI), we integrated the raw CT images, lesion regions, surrounding background, segmentation masks, and annotation overlays into the input terminal. This design enables the model to simultaneously capture multi-level visual cues—including internal tumor density, margin morphology, and reactive changes in the adjacent lung parenchyma—during a single forward pass. This closely mirrors the real-world diagnostic workflow of radiologists, who evaluate not only the primary mass but also lobulation, spiculation, pleural indentation, and surrounding tissue reactions. The lesion-level visualization further supports this interpretation. In representative cases, activation heatmaps showed that the high-response regions were mainly located within or around the segmented tumor area, rather than being distributed over unrelated lung regions or image background. This finding suggests that the deep representation model did not merely rely on global image appearance, but learned lesion-centered features together with peritumoral contextual information. Such visual evidence is consistent with the design rationale of the seven-channel CT input, which explicitly integrates the original CT image, lesion mask, overlay information and surrounding pulmonary background. Nevertheless, these activation maps should be interpreted as qualitative evidence of model attention rather than direct biological proof of mutation-specific imaging mechanisms. By structurally incorporating these contextual details into the feature extraction pipeline, MedicalDINOv3 may achieve more robust representation learning than models based solely on semantic CT features or handcrafted radiomics features. This interpretation is further supported by the ablation results, which showed that even in the absence of any auxiliary features, the deep representations derived solely from the multi-dimensional CT input architecture still consistently outperformed traditional machine learning models and purely semantic baseline models. In contrast, the baseline models revealed inherent limitations: RF relies on subjective semantic signs, SVM is constrained by the representational ceiling of handcrafted radiomics, and the Qwen branch underperformed in this specific classification task. This further confirms that relying solely on low-dimensional feature spaces or textual semantic mapping is insufficient to decode the complex five-class phenotypic variations of lung adenocarcinoma.

Delving into the classification performance across distinct molecular subtypes reveals notable heterogeneity. The identification of *EGFR* wild-type was the most robust, suggesting a relatively clear radiological boundary between the wild-type and *EGFR*-mutant cohorts. Furthermore, the two subtypes harboring concurrent *TP53* mutations maintained good discriminability. In particular, the “*EGFR* L858 R + *TP53*”category achieved high AUC values in both internal and external validations. This implies that such concurrent mutations may confer a more aggressive biological behavior or induce more pronounced remodeling of the tumor microenvironment, making their imaging representations easier for deep networks to capture (Zhang et al., 2025). Conversely, the classification boundaries between the two classic sensitive mutations (*EGFR* 19del and *EGFR* L858 R) remained partially overlapping. This is consistent with their highly similar clinical and radiological profiles, highlighting the technical bottleneck of relying strictly on morphological imaging to cleanly separate closely related driver gene subtypes. Notably, the AUC of SVM for the two *TP53* co-mutated subtypes approached that of MedicalDINOv3. This indicates that traditional radiomics may still provide useful complementary information for assessing *TP53*-related subgroups. However, in the present cohort, the deep representation model showed better overall stability and class balance than the semantic and radiomics-based models. We incorporated the Qwen model as a non-visual semantic baseline to evaluate whether structured clinical information and radiological semantic descriptions alone could support molecular subtype classification. The results revealed that semantic-only modeling was markedly limited compared with vision-based deep learning approaches, underscoring the essential contribution of CT visual representations to accurate *EGFR/TP53* molecular stratification.

External validation results further delineate the practical application boundaries of this model. In the binary *EGFR* classification task using a public dataset, the model achieved an AUC of 0.6666; in the multi-center five-class validation, the overall accuracy reached 75.5%. However, the AUC of 0.6666 in the public binary *EGFR* classification task mainly reflects limitations in cross-cohort generalization and should not be directly interpreted as evidence against the potential clinical applicability of the model in East Asian populations. In the multicenter five-class validation, although the raw overall accuracy reached 75.5%, this metric was substantially influenced by class imbalance, with *EGFR* wild-type cases accounting for approximately 61% of the external cohort compared with 44.8% in the internal test set. Therefore, the raw accuracy may overestimate model performance and is not directly comparable with the internal evaluation results. When assessed using distribution-independent metrics, including balanced accuracy, macro-averaged F1-score, and subtype-specific AUCs, the model demonstrated overall inferior performance in the external cohort compared with the internal test set. Specifically, the recognition rates for the wild-type and “*EGFR* L858 R + *TP53*”subtypes remained stable, whereas the discriminative capacity for “*EGFR* 19del” and its *TP53* co-mutated counterpart declined. Cross-cohort ROC analyses reveal that while the model retains baseline stratification capabilities, its overall performance experiences attenuation compared to the internal test set. This objective performance loss is primarily driven by inherent heterogeneities across different centers regarding data sources, scanning protocols, population demographics, and baseline molecular proportions. Consequently, the precise clinical positioning of the proposed CT prediction model should be an “auxiliary assessment tool” rather than an “alternative diagnostic method.” Its true clinical utility lies in rapid risk stratification during initial diagnosis, predictive assessment during the waiting period for genetic testing, and supplementary evaluation when tissue sampling is restricted (Saad et al., 2023; Gualandi et al., 2025).

This study has several limitations. As a single-center retrospective cohort study, despite the substantial sample size and the inclusion of external validation, selection bias is inevitable during internal data collection, image quality control, and case enrollment. Additionally, the sample distribution across the five classes is imbalanced. The relative scarcity of cases with concurrent *TP53* mutations limits the stability of the model’s deep parameter learning and increases the risk of statistical fluctuations when evaluating minority classes. Although class weighting, Focal Loss, and training-set oversampling strategies were implemented to mitigate this imbalance, these approaches could not completely eliminate the potential bias introduced by the underlying class distribution. Moreover, while the macro-averaged AUC provides a more balanced assessment by reducing the influence of class imbalance on overall performance evaluation, it may still obscure the actual predictive performance of minority subtypes. Therefore, clinical interpretation should consider subtype-specific AUC values in conjunction with global metrics to avoid misleading conclusions caused by reliance on a single evaluation criterion. Regarding external generalization, although an independent cohort was introduced, its imaging hardware parameters and population baselines differed from those of the internal cohort; this cross-domain data shift directly restricts the model’s generalizability. Finally, driven by the goal of developing a universally applicable and non-invasive clinical translation tool, the input source was restricted exclusively to CT images. The absence of high-dimensional information—such as histopathology, whole-genome sequencing, and longitudinal follow-up data—limits further breakthroughs in predictive efficacy. Future efforts will focus on refining the model’s robustness using multi-center, large-sample, and standardized imaging cohorts, while actively exploring the added value of fusing medical imaging with multimodal clinical phenotypes (Liu et al., 2025).

In conclusion, routine chest CT images contain objective visual information capable of characterizing *EGFR* mutation subtypes and concurrent *TP53* mutation status in lung adenocarcinoma. Compared to traditional semantic mapping and handcrafted radiomic pathways, the modeling architecture based on deep CT representations achieves superior global diagnostic efficacy and class balance in complex five-class tasks. Although cross-center generalizability requires further optimization, this study confirms that the proposed deep learning model holds clinical potential to provide a non-invasive, interpretable auxiliary assessment for the molecular profiling of lung adenocarcinoma.

Several limitations should be acknowledged. First, although all models were evaluated using an identical independent test cohort, uncertainty quantification and formal statistical comparison between models were not performed in the current study. Bootstrap confidence intervals and paired statistical tests may provide additional evidence regarding the reliability of model comparisons. Second, the number of patients in several molecular subgroups, particularly *TP53* co-mutated categories, was relatively limited, which may affect the stability of subtype-specific performance estimates. Future studies with larger multicenter cohorts and repeated validation strategies are warranted to further confirm the robustness and statistical reliability of the proposed framework.

## 5 Conclusion

This study developed a five-class prediction model based on pretreatment chest CT to stratify *EGFR* mutation subtypes and concurrent *TP53* mutations in lung adenocarcinoma. Our findings confirm that the MedicalDINOv3 model, driven by deep CT representation extraction, not only achieves optimal overall diagnostic efficacy in the internal test set but also maintains robust discriminative capacity across diverse molecular subtypes. Multicenter external validation further demonstrated that the model maintained feasible stratification capability when applied to cross-domain heterogeneous data from East Asian lung adenocarcinoma cohorts with standardized thin-slice contrast-enhanced CT acquisition. However, substantial variability was observed among molecular subtypes, with notable performance degradation in clinically relevant categories including *EGFR* 19del and *EGFR* 19del + *TP53*. These findings indicate that the current model has not yet achieved uniformly reliable clinical applicability across all *EGFR* mutation subtypes. Moreover, evaluation on the public Western cohort revealed limited cross-population generalizability, with an AUC of 0.6666 in *EGFR* binary classification, only marginally exceeding random discrimination, suggesting that the model should not yet be directly applied for independent molecular subtype determination. Future studies incorporating larger-scale, multi-ethnic, multi-device, and standardized multicenter imaging cohorts are warranted to improve domain adaptation, robustness, and clinical translational potential. Ultimately, routine chest CT images harbor discernible visual signatures that reflect the complex molecular profiling of lung adenocarcinoma. Modeling strategies centered on deep representation learning can provide robust, non-invasive imaging evidence for precise pre-treatment risk stratification and act as a supplementary assessment for molecular diagnostics.

## Data Availability

The raw data supporting the conclusions of this article will be made available by the authors, without undue reservation.
