# Supplementary material for: CT-based prediction of EGFR mutation subtypes and TP53 co-mutation status in lung adenocarcinoma
Source: Front Mol Biosci. 2026 Oct 21;13:1898841. doi: 10.3389/fmolb.2026.1898841 (PMC13730897; doi:10.3389/fmolb.2026.1898841)
Supplement: Supplementary file 1 [file Supplementaryfile1.docx]

2. **Supplementary information**

**Supplementary Table S1. Detailed baseline clinical characteristics stratified by molecular subtype**

| **Characteristic** | **Overall**  **(n=1633)** | **EGFR wild type**  **(n=731)** | **EGFR 19del**  **(n=295)** | **EGFR L858R**  **(n=399)** | **EGFR 19del+TP53**  **(n=118)** | **EGFR L858R+TP53**  **(n=90)** | **P value** |
| --- | --- | --- | --- | --- | --- | --- | --- |
| **Age, years, mean ± SD** | 63.75 ± 10.24 | 63.63 ± 10.97 | 62.77 ± 9.40 | 64.79 ± 9.79 | 61.16 ± 9.39 | 66.68 ± 8.62 | <0.001 |
| **Sex** |  |  |  |  |  |  | <0.001 |
| Male | 801 (49.1%) | 456 (62.4%) | 123 (41.7%) | 133 (33.3%) | 52 (44.1%) | 37 (41.1%) |  |
| Female | 832 (50.9%) | 275 (37.6%) | 172 (58.3%) | 266 (66.7%) | 66 (55.9%) | 53 (58.9%) |  |
| **Smoking status** |  |  |  |  |  |  | <0.001 |
| Never smoker | 1183 (72.4%) | 472 (64.6%) | 225 (76.3%) | 323 (81.0%) | 91 (77.1%) | 72 (80.0%) |  |
| Former smoker | 355 (21.7%) | 213 (29.1%) | 54 (18.3%) | 55 (13.8%) | 18 (15.3%) | 15 (16.7%) |  |
| Current smoker | 95 (5.8%) | 46 (6.3%) | 16 (5.4%) | 21 (5.3%) | 9 (7.6%) | 3 (3.3%) |  |
| **Family history of malignancy** |  |  |  |  |  |  | 0.261 |
| Yes | 83 (5.1%) | 29 (4.0%) | 14 (4.7%) | 28 (7.0%) | 7 (5.9%) | 5 (5.6%) |  |
| **Tumor laterality** |  |  |  |  |  |  | 0.005 |
| Left lung | 643 (39.4%) | 279 (38.2%) | 115 (39.0%) | 164 (41.1%) | 54 (45.8%) | 31 (34.4%) |  |
| Right lung | 952 (58.3%) | 432 (59.1%) | 172 (58.3%) | 233 (58.4%) | 63 (53.4%) | 52 (57.8%) |  |
| Bilateral | 38 (2.3%) | 20 (2.7%) | 8 (2.7%) | 2 (0.5%) | 1 (0.8%) | 7 (7.8%) |  |
| **Clinical stage** |  |  |  |  |  |  | <0.001 |
| I–III | 674 (41.3%) | 394 (53.9%) | 95 (32.2%) | 130 (32.6%) | 25 (21.2%) | 30 (33.3%) |  |
| IV | 959 (58.7%) | 337 (46.1%) | 200 (67.8%) | 269 (67.4%) | 93 (78.8%) | 60 (66.7%) |  |
| **Specimen source** |  |  |  |  |  |  | <0.001 |
| Percutaneous lung biopsy | 884 (54.1%) | 311 (42.5%) | 163 (55.3%) | 233 (58.4%) | 105 (89.0%) | 72 (80.0%) |  |
| Pleural/pericardial effusion cytology | 195 (11.9%) | 115 (15.7%) | 33 (11.2%) | 39 (9.8%) | 1 (0.8%) | 7 (7.8%) |  |
| Surgical specimen | 353 (21.6%) | 197 (26.9%) | 65 (22.0%) | 80 (20.1%) | 7 (5.9%) | 4 (4.4%) |  |
| Bronchoscopic biopsy | 116 (7.1%) | 55 (7.5%) | 22 (7.5%) | 29 (7.3%) | 4 (3.4%) | 6 (6.7%) |  |
| Lymph node/mediastinal sampling | 66 (4.0%) | 49 (6.7%) | 7 (2.4%) | 8 (2.0%) | 1 (0.8%) | 1 (1.1%) |  |
| Other metastatic-site biopsy | 19 (1.2%) | 4 (0.5%) | 5 (1.7%) | 10 (2.5%) | 0 (0.0%) | 0 (0.0%) |  |
| CEA, ng/mL, median (IQR) | 5.45 (1.96–35.94) | 3.37 (1.43–16.61) | 8.63 (2.54–54.90) | 8.17 (2.63–42.23) | 8.62 (3.45–87.46) | 13.71 (2.71–105.78) | <0.001 |
| **Lymph node metastasis** |  |  |  |  |  |  | <0.001 |
| Yes | 1059 (64.8%) | 356 (48.7%) | 233 (79.0%) | 320 (80.2%) | 92 (78.0%) | 58 (64.4%) |  |
| **Bone metastasis** |  |  |  |  |  |  | <0.001 |
| Yes | 478 (29.3%) | 153 (20.9%) | 106 (35.9%) | 137 (34.3%) | 55 (46.6%) | 27 (30.0%) |  |
| **Intrapulmonary metastasis** |  |  |  |  |  |  | <0.001 |
| Yes | 427 (26.1%) | 104 (14.2%) | 107 (36.3%) | 140 (35.1%) | 44 (37.3%) | 32 (35.6%) |  |
| **Brain metastasis** |  |  |  |  |  |  | <0.001 |
| Yes | 255 (15.6%) | 60 (8.2%) | 61 (20.7%) | 79 (19.8%) | 35 (29.7%) | 20 (22.2%) |  |
| **Malignant pleural effusion** |  |  |  |  |  |  | <0.001 |
| Yes | 345 (21.1%) | 99 (13.5%) | 80 (27.1%) | 118 (29.6%) | 31 (26.3%) | 17 (18.9%) |  |
| **Liver metastasis** |  |  |  |  |  |  | <0.001 |
| Yes | 105 (6.4%) | 28 (3.8%) | 29 (9.8%) | 21 (5.3%) | 22 (18.6%) | 5 (5.6%) |  |
| **Adrenal metastasis** |  |  |  |  |  |  | 0.321 |
| Yes | 95 (5.8%) | 36 (4.9%) | 22 (7.5%) | 28 (7.0%) | 6 (5.1%) | 3 (3.3%) |  |

This table provides the full baseline clinical statistics of the study cohort across the five molecular subgroups, including demographic characteristics, smoking status, tumor laterality, clinical stage, specimen source, serum CEA level, and metastatic status.

**Supplementary Table S2. Class-specific AUC values of different models for the five molecular subtypes**

| **Molecular subtype** | **MedicalDINOv3** | **MedicalDINOv3-CT only** | **MedicalDINOv3-Clinical text only** | **SVM** | **Random Forest** | **Qwen** |
| --- | --- | --- | --- | --- | --- | --- |
| **EGFR wild type** | 0.917 | 0.882 | 0.759 | 0.808 | 0.845 | 0.719 |
| **EGFR 19del** | 0.855 | 0.676 | 0.582 | 0.712 | 0.794 | 0.646 |
| **EGFR L858R** | 0.894 | 0.807 | 0.654 | 0.755 | 0.826 | 0.688 |
| **EGFR 19del + TP53** | 0.854 | 0.406 | 0.666 | 0.814 | 0.840 | 0.661 |
| **EGFR L858R + TP53** | 0.873 | 0.403 | 0.490 | 0.870 | 0.789 | 0.783 |

This table summarizes the one-vs-rest AUC values of MedicalDINOv3, support vector machine, random forest, and Qwen-based models for EGFR wild type, EGFR 19del, EGFR L858R, EGFR 19del + TP53, and EGFR L858R + TP53 in the internal test set.

**Supplementary Fig. S1 Receiver operating characteristic curves of the four models in the five-class classification task.**

**D**

**C**

**B**

**A**

**Fig. S1** Comparison of ROC curves of the four models in the five-class stratification of EGFR and concurrent TP53 mutations in lung adenocarcinoma. **A** ROC curves of the MedicalDINOv3 model; **B** ROC curves of the Qwen model; **C** ROC curves of the random forest (RF) model; **D** ROC curves of the support vector machine (SVM) model.

This figure shows the overall ROC performance of MedicalDINOv3, support vector machine, random forest, and Qwen-based models in the five-class classification task and complements the class-specific ROC analysis shown in Fig. 5.

**Supplementary Fig. S2 Class-wise precision, recall, and F1 comparisons of the four models in the five-class classification task.**

This figure presents class-wise precision, recall, and F1 scores for the four models, providing a more detailed comparison of class-specific classification performance beyond the overall metrics reported in Table 2.

**A**

**B**

**C**

**D**

**Fig. S2** Comparison of classification metrics among the four models across molecular subtypes of EGFR and concurrent TP53 mutations in lung adenocarcinoma. **A** MedicalDINOv3 model (precision, recall, and F1-score); **B** Qwen model (precision, recall, and F1-score); **C** Random forest (RF) model (precision, recall, and F1-score); **D** Support vector machine (SVM) model (precision, recall, and F1-score).
